# Health-related quality of Life in patients with chronic hepatitis C receiving Sofosbuvir-based treatment, with and without Interferon: a prospective observational study in Egypt

**DOI:** 10.1186/s12876-017-0581-1

**Published:** 2017-01-21

**Authors:** Naglaa F. A. Youssef, Mohamed El Kassas, Amany Farag, Ashley Shepherd

**Affiliations:** 1 Faculty of Nursing, Cairo University, Cairo, 11562 Egypt; 2 Faculty of Medicine, Helwan University, Cairo, Egypt; 3 Faculty of Health Sciences and Sport, University of Stirling, Stirling, FK9 4LA Scotland, UK

**Keywords:** Directly acting antivirals-DAAs, Health related quality of life, Social support, Patient reported outcomes (PROs), Hepatitis C Virus antiviral therapy

## Abstract

**Background:**

The Egyptian government introduced the first directly acting antivirals (DAAs) into Egypt through the government funded National Treatment Program. As yet, there has been no investigation into the effects of these new DAAs therapies on patient reported outcomes (PROs). This study aimed to (1) assess the PROs (health-related quality of life (HRQoL), mental health and perceived social support) of HCV patients receiving DAAs therapy prior, during and at the end of therapy; (2) evaluate PROs of Interferon-free (dual) users versus Interferon-containing (triple) users cross the three different time periods; and (3) identify the predictors of HRQoL of DAAs therapy users cross the three different time periods.

**Methods:**

A prospective observational design was used. Patients with chronic HCV undergoing treatment following the Egyptian National Guidelines at one of the national treatment centers were approached. Data collection occurred in the period from February to October 2015. Data was collected at three time points: (1) baseline (time 0: T0), before initiating therapy); (2) 5/6 weeks after initiation of therapy (time 1 of therapy: T1) and at the end of the therapy (Time 2: T2). Four PROs questionnaires were utilized for data collection: (1) Multidimensional Scale of Perceived Social Support (MSPSS), (2) The Depression Anxiety Stress Scales (DASS-21), (3) the Liver Disease Symptom Index-2.0 (LDSI-2.0) for testing disease specific HRQoL and (4) the Center for Adherence Support Evaluation (CASE) Index, alongside the background data sheet.

**Results:**

Sixty-two patients participated. There was a change in HRQoL, symptom experience and mental health across the three different time periods. HRQoL was impaired more after starting the course of therapy (T1) than at baseline (T0) and end of therapy (T2), z ≥ -2.04, *p* ≤ .04. Also, symptom experience deteriorated more during the treatment period than at the baseline, Z ≥ -1.97, *p* ≤ .04. Anxiety and stress were significantly higher during the treatment period than at the end of treatment. Perceived social support was significantly higher during the treatment period than at baseline and end of therapy, Z ≥ -2.27, *p* ≤ .023. During the course of therapy, triple users were more likely to report poorer HRQoL and anxiety than dual users (*p* ≤ .04). By the end of therapy, the two arms of therapy had no significant differences in any of the PROs.

At baseline, the predictor model significantly (*p* = .000) explained 37.5% of the variation in the HRQoL prior to therapy. Depression was the main variable that contributed to (41.3%) predicting change in HRQoL prior to therapy. During therapy, the model significantly (*p* = .000) explained 76% of the variation in the HRQoL-T1. Stress-T1, body mass index (BMI)-T1 and HRQoL-T0 significantly and respectively predicted 44.4, 46.5 and 31.1% of the variation in HRQoL-T1. At the end of therapy, the model significantly (*p* = .000) predicted 80.5% of the variation in the HRQoL-T2. HRQoL-T1 and anxiety-T2 significantly predicted 72.3 and 61.6% of the variation in HRQoL-T2.

**Conclusions:**

Baseline HRQoL, depression and BMI should be systematically assessed before starting the antiviral therapy for early detection and the improvement of the impairment before the initiation of therapy. Anxiety should be frequently assessed and followed up through the course of antiviral therapy. The triple group required more nursing and practitioner attention due to increased anxiety levels and impaired HRQoL during the treatment therapy.

## Background

Worldwide, the prevalence of the hepatitis C virus (HCV) is 2.8%, causing a considerable global burden of morbidity and mortality [1, 2]. According to a nationally representative survey carried out in 2008 [3], Egypt has the highest HCV prevalence in the world; with 14.7% sero prevalence. Hepatitis C has been associated with substantial resource utilization as a result of its effect on the liver as well as other organ systems (the extrahepatic manifestations of HCV) [4, 5]. It has been widely reported to have a profound negative impact on patient’s health-related quality of life (HRQoL) because of the associated complications of advanced liver disease (i.e. encephalopathy, variceal hemorrhage, ascites) [6]. Also, employees being treated for hepatitis C had high rates of absenteeism and impairment of their work productivity [7]. HCV affects HRQoL through other avenues, such as fatigue, persistent flu-like symptoms, joint pain, itching, sleep disturbances, appetite changes, nausea, and depression [8, 9].

Patient reported outcomes (PROs) have become increasingly important data in clinical research, since they can provide the most complete assessment of the impact of chronic hepatitis C and its treatment on patients’ health status [10]. PROs have been defined as measurements that are based on reports that come directly from the patients about their health status without any amendment or interpretation by healthcare providers [11]. There are a number of important PROs that provide insight into patients’ experiences such as HRQoL. HRQoL has been defined as the patients’ subjective perception of the impact of their disease and/or its treatment on their daily life, and their physical, psychological and social functioning. These definitions clearly acknowledge that HRQoL is a multidimensional concept. Therefore, it has been considered the gold standard to measure patients’ experiences with their disease and treatment [12].

A number of antiviral therapies have been developed. Antiviral therapies can eradicate the virus resulting in improvements in liver histology, which prevents liver-related mortality [13] and enhances HRQoL because of symptoms’ alleviation and an increase in associated economic and social benefits. For example, work force participation and removal of social stigma can follow after successful treatment [13–15]. However, the toxicity associated with antiviral treatments can negatively affect HRQoL by way of diminished physical, emotional and social functioning [7, 16].

A pegylated interferon (peg-IFN)-based regimen has long been the standard treatment for patients with HCV [17]. Treatment with a peg-IFN-containing regimen has been shown to severely impair all PROs including work productivity, leading to negative patient experiences and lower adherence to the treatment regimen [7, 16].

The arrival of direct acting antivirals (DAAs), such as bocepreveir and telaprevir, in triple combination with peg-IFN and ribavirin, increased HCV clearance rates but might cause adverse effects that can further decrease HRQoL [18, 19]. Over the past few years, the treatment landscape for HCV has been changing rapidly, leading to the introduction of newer and improved, DAAs. Even in patients who were considered difficult to treat; these agents have reduced toxicity, increased barriers to resistance and led to reduced side effects [20–23] such as fatigue and neuropsychiatric problems [24]. The interferon-free treatment (dual) can potentially provide a number of important advantages; including higher efficacy, lower side effects and shorter duration of treatment, which can substantially increase adherence level and improve PROs [25].

The Egyptian government introduced sofosbuvir as the first DAAs into Egypt through the government funded National Treatment Program [26]. The first DAAs regimen used in the country was sofosbuvir, which was administered either as a triple therapy in combination with peg-IFN and ribavirin or as a dual therapy combined only with ribavirin (for interferon non-eligible patients). As of July 2015, other treatment options, including an all DAAs therapy with simepravir and sofosbuvir were introduced, with many more DAAs containing therapies soon to be approved [26].

To date, there have been no investigations into how Egyptian HCV patients receiving the new DAAs perceive their HRQoL. Therefore, this study is the first to evaluate a number of PROs, including health-related quality of life (HRQoL), mental health status (i.e. depression, anxiety and stress), and perceived social support among HCV patients, while receiving DAAs therapy; either an interferon-free or interferon-containing *regimen.*

Specifically this study aimed to:

1. Assess the PROs (i.e. health-related quality of life, mental health and perceived social support) of HCV patients receiving direct acting antivirals (DAAs) therapy prior, during and at the end of therapy.
2. Evaluate PROs of Interferon-free (dual) users versus Interferon-containing (triple) users prior, during and at the end of treatment.
3. Identify the predictors of HRQoL of DAAs therapy users prior, during and at the end of therapy.

## Methods

### Study design

A prospective observational design was used to conduct this study. Patients with chronic HCV being treated, using the Egyptian National Guidelines at one of the national treatment centres, were approached to participate. According to the most updated treatment protocol, patients undergo therapy for a relatively short period (3 to 6 months).

### Study population

A total of 80 patients were invited to participate in the study in the period from February to October 2015. Patients were randomly selected from HCV patients visiting the HCV specialized clinic. Patients were eligible to participate in the study if they met the following criteria:

- Had no significant psychiatric illnesses (diagnosed by psychiatrist),
- Aged 18 years or older,
- Eligible for starting DAAs therapy and
- Gave written consent to participate.

### Data collection

This study was conducted at the Outpatients clinic of the National Hepatology and Tropical Medicine Research Institute (NHTMRI), Cairo, Egypt. This institute is one of 42 specialized national treatment centres for treatment of viral hepatitis distributed throughout the country; and it is the largest of all these centres. This institute serves patients from different regions in Egypt and of various socioeconomic status. All the centres follow the same set of national guidelines for the treatment of patients with chronic HCV and are supervised by the National Committee for Control of Viral Hepatitis.

Data was collected at three points in time: (1) baseline [time 0 (T0), before initiating therapy]; (2) 5^th^ or 6^th^ week after initiation of therapy [time 1 (T1) of therapy] and (3) at the final week of therapy [time 2 (T2)]. At time 0, the patients were first interviewed following an appointment with the consultant in the outpatients’ clinic, where it was confirmed that they were eligible to start the HCV therapy.

#### Protocol of therapy

According to the 2014 Egyptian HCV national patient treatment guidelines, antiviral therapy was administered either as Interferon-free (dual) or Interferon-containing (triple) therapies. The recommended regimen for patients who were not eligible to receive peg-IFN (dual group) was daily Sofosbuvir (400 mg) plus weight-based RBV (1000 mg [<75 kg] to 1200 mg [>75 kg]) for 24 weeks. Inclusion criteria for treatment of patients who would be treated with Interferon free regimen was defined with the presence of any or all the following;

- Child score up to 8
- Total bilirubin ≤ 5
- Albumin ≥ 2.5
- Platelet count ≥ 30,000
- Prtothrombin concentration ≥ 50%
- Hemoglobin concentration ≥ 10 mg

Patients who were eligible to receive Interferon (triple group) would be treated with daily Sofosbuvir (400 mg) and weight-based RBV (1000 mg [<75 kg] to 1200 mg [>75 kg]) plus weekly Peg-INF for 12 weeks.

### Collection data procedure

#### Questionnaires and data collection

Four instruments were utilized for data collection alongside the background data sheet. Participants were interviewed face to face for around 30–40 min each, based on the patient co-operation and literacy level, to complete all the questionnaires.

The Multidimensional Scale of Perceived Social Support (MSPSS) [27] is a commonly used instrument for measuring the perceived adequacy of social support from three specific sources: family, friends and significant others [26]. Each subscale has four items that are rated on a seven point scale in the English version or on a three point scale in the Arabic version. The MSPSS can be computed to give the total and subscale scores for each of the three sources of support. The total score and subscale scores are calculated by adding up the participant’s responses. An increasing score represents increasing perceived adequacy of social support. This is the most appropriate tool for measuring perceived social support among patients waiting for OHS for many reasons. (1) It is the shortest and simplest tool available (12 items); (2) An Arabic version of MSPSS is available [27] and has been widely used among Arabic speaking people [26]. It has a high construct validity and internal consistency reliability with Cronbach’s alpha for total MSPSS = 0.74 [27].

The Depression Anxiety Stress Scale (DASS-21) [28] was used to assess mental health. The DASS-21 questionnaire includes 21 questions that measure anxiety, stress and depression separately, where each scale has seven questions. Each question is scored on a 4-point combined severity/frequency scale over the past week. The score ranges from 0 (did not apply to me at all) to 3 (applied to me very much, or most of the time). The overall score ranges from 0 to 21. Scores 0–4, 0–3 and 0–7 show normal levels of depression, anxiety and stress respectively; scores 5–7, 4–5 and 8–9 show low levels of depression, anxiety and stress respectively; scores of 8–11, 5–7 and 10–13 show moderate levels of depression, anxiety and stress respectively; scores 12–15, 8–9 and 14–17 show severe levels of depression, anxiety and stress respectively; and scores of 25+, 10+ and 18+ show extreme severe levels of depression, anxiety and stress respectively. Scores for depression, anxiety and stress are calculated by adding the scores for the relevant items. The Arabic version of this tool has been psychometrically validated.

The Liver Disease Symptom Index (LDSI)-2.0 is a short and psychometrically tested disease specific HRQoL questionnaire, which has been widely used with patients at different stages of a chronic infection with HCV [29, 30]. It is available in Arabic [29]. The LDSI-2.0 has two subscales that are used to assess symptom severity, and the impact of these symptoms on patients’ daily activities (symptom hindrance). The participants were asked if a symptom was experienced during the past week. If yes, the participants were asked to what extent it was affecting their daily lives and social contacts on a five-point Likert scale, with 0 = not at all, and 4 = to a high extent. Possible scores for each subscale ranged from 0 to 60 for the severity dimension and 0–36 for hindrance dimension. A higher score on the severity dimension represents a higher perception of the symptoms’ severity, and a higher score on the hindrance dimension represents a higher perception of the limitations of daily activities because of these symptoms. It also provides an overall total score that represents a disease specific HRQoL [29, 30].

The Center for Adherence Support Evaluation (CASE) Index [31] is a valid, reliable, simple and easy to administer instrument that measures self-reported antiretroviral therapy adherence. It is composed of three questions: Question 1: Self-reported frequency of ‘difficulty taking HCV medications on time’, with responses being: never, rarely, most of the time or all of the time. Question 2: Self-reported ‘average number of days per week at least one dose of HCV medications was missed’, with responses being: every day, 4–6 days per week, 2–3 days per week, once a week, less than once a week or never. Question 3: Self-reported ‘last time missed at least one dose of HCV medications’, with responses being: within the past week, 1–2 weeks ago, 3–4 weeks ago, between 1 and 3 months ago, more than 3 months ago or never. The total index score (INDEXSCORE) > 10 indicates good adherence, while < 10 indicates poor adherence.

Socio-demographic and medical data sheets were designed by the researchers and divided into two parts: (i) the socio-demographic sheet was used to collect data related to the participants’ characteristics; such as age, gender, employment status, occupation, education level, marital status and medical history, and (ii) the medical data sheet was used to record the diagnosis, disease duration, type of antiviral therapy, comorbidity (i.e. diabetes, hypertension), and other factors. The Body Mass Index (BMI) was calculated using the standard formula: BMI = kg/m^2^.

### Statistical methods

The Statistical Package for the Social Sciences 20 (IBM SPSS, Armonk, New York, United States) was used for the data analysis. Descriptive statistics were computed to summarize data. Individual variables were examined by percentages, means, and SDs. A non-parametric statistical technique, such as chi-square for independence, was used to compare the frequencies of nominal variables. Differences among the two arms of therapy were examined by independent *t* test. Friedman test was used to examine the change in HRQoL, mental health and social support at the three time points. Wilcoxon Signed Rank Test (Post-hoc pairwise) with Bonferroni correction was performed for HRQoL, mental health and social support across the three time points. A multiple linear regression analysis using “stepwise forward method” was used to investigate the factors associated with HRQoL. Since this was an exploratory study, there was no prior decision regarding the order of entering the variables in the model [32]. The multiple regression assumptions were investigated and there was no violation of normality, linearity, and multicollinearity. All statistical analyses were two tailed with *p* < .05 as the significance level.

## Results

### Characteristics of the participants

**Fig. 1:**
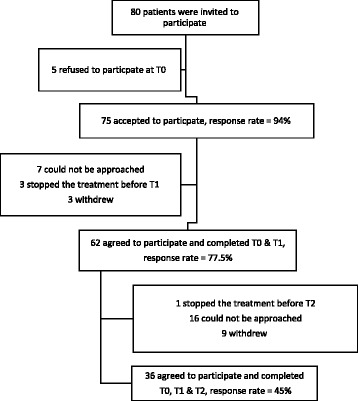
Sample flow diagram

Sixty-two patients in total participated in this study at T0 and T1; and 36 participated at T2 (Fig. 1). The baseline demographic and medical characteristics revealed that the mean age of the sample (*n* = 62) was 54.06 ± [standard deviation (SD) 10.41 years] (Table 1). The sample contained an almost equal number of males (48.4%) and females (51.6%). Most of the sample were married (72.6%), and employed (64.3%). There was an equal distribution between cirrhotic and non-cirrhotic participants, with disease discovery duration ranging from 3 to 216 months. Half of the participants had a medical comorbidity, with diabetes (30.6%) and hypertension (24.2%) the most commonly reported comorbidities (Table 1). About 87.1% of the patients were treatment naïve which meant they had never received HCV antiviral therapy before this study. Experienced patients had previously received HCV antiviral therapy (peg-IFN).

**Table 1:** Baseline demographic and clinical characteristics of the participants

	Number (total 62)	Percent
Age	54.06 ± 10.41*	
Gender		
• Males	30	48.40
• Females	32	51.60
Education		
• Uneducated	24	38.70
• Can read and write or preparatory	12	29.00
• Secondary	12	19.40
• University	8	12.90
Marital status		
• Unmarried	17	27.40
• Married	45	72.60
Employment status		
• Unemployed	17	27.40
• Employed	17	27.40
• Housewife	28	45.20
Residence area		
• Rural	15	24.20
• Urban	47	75.80
Diseases stage		
• Non cirrhotic	31	50
• Cirrhotic	31	50
Number of comorbidities		
• 0	31	50
• 1	17	27.4
• 2	11	17.7
• 3	3	4.8
Type of comorbidity		
• Hypertension	15	24.20
• Diabetes	19	30.60
• Others (renal disease, disc, heart disease, asthma, peptic ulcer)	11	17.70
Disease duration (Range from 3 to 216 months)	71.10 ± 57.72*	
Type of treatment		
• Dual	31	50
• Triple	31	50
Treatment experience		
• Naive	54	87.10
• Experienced	8	12.90
Treatment paying method		
• Self	6	9.70
• Governmental	56	90.30
BMI		
T0	30.6 ± 4.24*	
T1	30.4 ± 4.53*	
PCR at T1 (50 cases)		
• Negative	32	64
• Positive	18	36

*BMI* body mass index, *PCR* Polymerase Chain Reaction, *T0* Time 0, *T1* time 1

*Mean ± SD

*P value: * ≤ .05 at two tailed

### Comparison between males and females

A comparison between males (*n* = 30) and females (*n* = 32) demographic and medical data at baseline and their PROs through the three time points was conducted. Only significant results are presented in Table 2. As has been observed, females were more likely to be older and obese than males. Prior to therapy, females had a poorer HRQoL, higher levels of symptoms’ severity, hindrance of symptoms in their daily life, and a higher level of depression, than males. During the course of therapy, depression remained higher among females than males. At the end of therapy, there was no significant difference between males’ and females’ PROs.

**Table 2:** Comparison between males and females

	Mean ± Std. Deviation	t
Age		
• Male	49.48 ± 12.19	-3.68**
• Female	58.34 ± 5.92	
PCR-T0		
• Male = 28	890248.43 ± 1049454.60	2.22*
• Female = 28	411320.44 ± 451668.92	
BMI-T0		
• Male	29.10 ± 3.86	-2.83**
• Female	31.99 ± 4.15	
MBI-T1		
• Male	28.88 ± 3.83	-2.74**
• Female	31.88 ± 4.71	
HRQoL-T0		
• Male	23.73 ± 15.89	-3.43**
• Female	39.06 ± 19.66	
Symptom Severity-T0		
• Male	18.53 ± 10.30	-3.12**
• Female	27.43 ± 12.28	
Symptom Hindrance-T0		
• Male	5.20 ± 6.66	-3.27**
• Female	11.62 ± 8.57	
Depression-T0		
• Male	5.56 ± 5.88	-3.52**
• Female	11.47 ± 7.19	
Depression-T1		
• Male	7.20 ± 6.46	-2.32*
• Female	11.28 ± 7.69	

Note: only significant results are presented, sample of male = 30 & female = 32

*PCR-T0* Polymerase Chain Reaction time0, *BMI-T0* body mass index time 0, *BMI-T1* body mass index time 1, *HRQoL-T0* health-related quality of life time 0

*P value: * ≤ .05, ** ≤ .001, *** ≤ .0001 at two tailed

### Objective 1: PROs (health-related quality of life, mental health and perceived social support) of HCV patients receiving DAAs therapy prior, during and at the end of therapy

Friedman test for repeated measures was computed, among the patients who participated at the three different time points (*n* = 36), to compare HRQoL [LDSI-2.0 total score], symptom experience [two subscales of LDSI-2.0: severity and hindrance], mental health [depression, anxiety and stress], and perceived social support across the three different time points (Table 3).

**Table 3:** PROs of HCV patients receiving DAAs therapy prior, during and at the end of therapy

Variables/Time			Friedman test		Wilcoxon test		
	N	Mean + SD	Chi-Square	*P*-value	T	Z	*P* value (r)
HRQoL							
T0	36	31.02 ± 15.30	8.35	.01	T0 - T1 = 436	-2.37	.01 (-.30)
T1	36	39.63 ± 21.11					
T2	36	34.50 ± 19.96			T1 –T2 = 178.50	-2.04	.04(-.26)
Symptom experience							
Symptom severity							
T0	36	23.19 ± 10.21	5.41	.06	T0 - T1 = 458	-1.97	.04(-.25)
T1	36	27.36 ± 13.07					
T2	36	24.25 ± 12.75					
Symptom hindrance							
T0	36	7.83 ± 6.40	9.89	.007	T0 - T1 = 358	-2.58	.01(-.33)
T1	36	12.27 ± 9.00					
T2	36	10.25 ± 7.89					
Mental Health							
Depression							
T0	36	8.91 ± 7.25	8.79	.01	T0 - T1 = 385	-2.28	.02(-.29)
T1	36	10.77 ± 7.18					
T2	36	8.97 ± 6.57					
Anxiety							
T0	36	7.33 ± 5.80	6.82	.03	T0 - T1 = 379	-2.57	.01(-.33)
T1	36	8.88 ± 5.87					
T2	36	6.86 ± 6.05			T1 - T2 = 80	-2.63	.009(-.34)
Stress							
T0	36	10.94 ± 7.35	4.86	.08	T1 - T2 = 103.50	-2.47	.01(-.32)
T1	36	12.05 ± 6.83					
T2	36	9.75 ± 6.80					
MSPSS							
T0	36	2.41 ± .47	11.35	.003	T0 - T1 = 212	-2.27	.02(-.29)
T1	36	2.51 ± .37					
T2	36	2.41 ± .39			T1 - T2 = 146	-2.77	.006(-.36)

*HRQoL* Health-related quality of life time 0, *T0* time 0, *T1* time 1, *T2* time 2

*P* value significant at two tailed

There was a significant effect for time on HRQoL, *X* ^2^ = 8.35, *p* = .015. A Wilcoxon Signed Rank Test with Bonferroni correction was performed between each of the time points with r indicating the effect size based on the Cohen criteria. A Wilcoxon Signed Rank Test revealed there was a statistically significant impairment of HRQoL after starting the course of therapy (T1) than at baseline (T0) and end of therapy (T2), z ≥ -2.04, *p* ≤ .04, with a medium effect size (*r* ≥ -.26) (Table 3).

Also, there was a significant effect for time (T0 vs. T1) on symptom experience (severity & hindrance) and mental health (depression & anxiety), (*X* ^2^ = 5.41, 9.89, 8.79, 6.82 respectively, *p* < .05). A Wilcoxon Signed Rank Test revealed there was a statistically significant impairment of symptom experience and mental health during the treatment period (T1, 5/6 weeks) than at the baseline, Z ≥ -1.97, *p* ≤ .04, with a medium effect size (*r* ≥ -.25). Additionally, anxiety and stress were significantly higher during the treatment period than at the end of treatment. Perceived social support was also significantly higher during the treatment period than at baseline (T0) and end of therapy (T2), Z ≥ -2.27, *p* ≤ .023, with a medium effect size (*r* ≥ -.29) (Table 3).

### Comparison of basic demographic and medical data between dual and triple users

For the purpose of comparing dual and triple therapy users, the baseline demographic and medical characteristics of the two groups of participants were initially compared using the nonparametric statistical tests (i.e. Chi-square test and independent-samples Mann-Whitney *U* Test). Dual participants were more likely to live in urban areas (Pearson Chi-Square = 7.123, *p* = .02) and more cirrhotic (Pearson Chi-Square = 7.806, *p* = .01) than triple therapy participants. However, the rates of baseline factors that could potentially impact HRQoL-T1 (i.e. age, gender, employment status, depression-T0, anxiety-T0, stress-T0, disease duration, PCR-T0, BMI-T0, symptom severity-T0, symptom hindrance-T1 and HRQoL-T0) were similar between the two arms of therapy (all *P* ≥ .05) (Table 4).

**Table 4:** Comparison of basic demographic and medical data between dual and triple users

Variables	Type of therapy		*P* value Sig. (2-tailed)
	Dual (*n* = 31)	Triple (*n* = 31)	
Gender			
• Male	12	18	.20
• Female	19	13	
Education			
• Uneducated	17	7	.02
• Educated	14	24	
Employment			
• Employed	6	11	.24
• Unemployed	8	9	
• Housewives	17	11	
Martial status			
• Single	8	9	.50
• Married	23	22	
Area of residence			
• Rural	3	12	.02
• Urban	28	19	
Treatment experience			
• Naïve	30	24	.05
• Experience	1	7	
Diagnosis			
• Non-cirrhotic	10	21	.01
• Cirrhotic	21	10	
Mean ± SD			
Age			
	56.47 ± 9.32	51.65 ± 11.03	.07
Disease duration (months)			
	81.71 ± 62.86	60.48 ± 50.94	.15
PCR-T0			
	655845.81 ± 997681.03	645348.15 ± 638028.61	.96
PCR-T1 (50 cases)			
• Negative 32 (51.6%)			
• Positive 18 (29.0%)			

*PCR-T0* Polymerase Chain Reaction time 0, *PCR-T1* Polymerase Chain Reaction time 1

*P* value significant at two tailed

### Comparison of HRQoL, mental health and symptom experience between dual and triple users

Table 4 presents the comparison of PROs (i.e. HRQoL, mental health and symptom experience) between the dual and triple therapy users at the three points in time. During the course of therapy (after 5/6 weeks), triple users (*n* = 31) were more likely to report poorer HRQoL and anxiety than dual users (*n* = 31) (*p* ≤ .04). Using Pearson correlation showed that there is a high significant association between anxiety and HRQoL during the treatment period (r - = .78, *p* = .000). Anxiety during the time period was significantly correlated with baseline HRQoL (r = .51. *p* = .000). By the end of therapy, the two arms of therapy had no significant differences in any of the PROs (Table 5). A post hoc power calculation for the Mann Whitney *U* test of differences in HRQoL between the dual and triple therapy group during treatment is estimated power of 0.33.

**Table 5:** PROs prior, during and at the end of treatment among dual and triple users

	Type of therapy		Independent-samples Mann-Whitney *U* Test
	Dual *N* =31	Triple *N* = 31	*P* value Sig. (2-tailed)
Baseline	X ± SD		
BMI-T0	31.26 ± 4.16	29.92 ± 4.26	.31
HRQoL-T0	32.54 ± 21.23	30.74 ± 17.67	.85
Symptom Severity-T0	23.32 ± 13.14	22.93 ± 11.24	.97
Symptom Hindrance-T0	9.22 ± 9.09	7.80 ± 7.50	.64
MSPSS-T0	2.22 ± .57	2.49 ± .39	.05
Depression-T0	9.12 ± 7.51	8.09 ± 6.92	.59
Anxiety-T0	6.41 ± 5.83	7.51 ± 6.03	.53
Stress-T0	8.64 ± 6.61	10.80 ± 7.63	.29
After 5/6 weeks	X ± SD		
BM-IT1	31.24 ± 4.47	29.60 ± 4.50	.16
HRQoL-T1	31.06 ± 24.06	41.54 ± 21.62	.04
Symptom Severity-T1	21.77 ± 15.09	28.51 ± 12.72	.05
Symptom Hindrance-T1	9.29 ± 9.64	13.03 ± 9.67	.09
MSPSS-T1	2.34 ± .55	2.54 ± .38	.23
Depression-T1	9.12 ± 7.67	9.48 ± 7.15	.72
Anxiety-T1	5.64 ± 5.77	9.19 ± 6.16	.01
Stress-T1	9.00 ± 7.46	11.87 ± 7.09	.11
INDEXSCORE-T1	15.74 ± .77	15.45 ± 1.45	.87
At the end of therapy	X ± SD		
HRQoL-T2	26.60 ± 17.01	37.53 ± 20.47	.15
Symptom Severity-T2	18.20 ± 12.50	26.57 ± 12.29	.06
Symptom Hindrance-T2	8.40 ± 5.10	10.96 ± 8.72	.52
MSPSS-T2	2.40 ± .40	2.41 ± .40	.84
Depression-T2	6.20 ± 6.49	10.03 ± 6.40	.10
Anxiety-T2	4.50 ± 3.53	7.76 ± 6.61	.27
Stress-T2	6.40 ± 5.16	11.03 ± 6.99	.08
INDEXSCORE-T2	15.90 ± .31	15.30 ± 1.46	.41

*BMI-T0* body mass index time 0, *HRQoL-T0* health-related quality of life time 0, *MSPSS-T0* Multidimensional Scale of Perceived Social Support time 0, *BMI-T1* body mass index time 1, *HRQoL-T1* health-related quality of life time 1, *MSPSS-T1* Multidimensional Scale of Perceived Social Support time 1, *INDEXSCORE-T1* The total index score of the Center for Adherence Support Evaluation (CASE) Index time 1, *HRQoL-T2* health-related quality of life time 2, *MSPSS-T2* Multidimensional Scale of Perceived Social Support time 2, *INDEXSCORE-T2* The total index score of the Center for Adherence Support Evaluation (CASE) Index time 2

*P* value significant at two tailed

### Objective 2: PROs of dual users versus triple users prior, during and at the end of treatment

The pattern of change in PROs among triple (*n* = 26) and dual (*n* = 10) therapy users prior, during and at the end of therapy was considered using Friedman test (Table 6). Results revealed that the triple therapy group had significantly poorer HRQoL, symptom severity, symptom hindrance and depression, and more increase in perceived social support score at T1 than at baseline, (Chi-Square ≥ 6.653, *p* ≤ .036). In contrast, dual users had no significant change in their HRQoL, symptom experience and mental health (Chi-Square ≤ 5.706, *p* ≥ .058), while their perceived social support was significantly increased during T1 in comparing to T0 and T2 (*p* = .037) like triple users (Table 6). A post hoc power calculation for the Friedman test of change in HRQoL in the dual group over time is estimated power between 0.21 and 0.41.

**Table 6:** Pattern of change in PROs among dual and triple users prior, during and at the end of treatment

Variables/Time	Triple therapy *N* = 26			Dual therapy *N* = 10		
	Mean + SD	Chi-Square	*P*-value	Mean + SD	Chi-Square	*P*-value
HRQoL						
T0	29.81 ± 15.87	10.26	.006	34.20 ± 13.98	.47	.79
T1	43.35 ± 21.15			30.00 ± 18.63		
T2	37.54 ± 20.47			26.60 ± 17.02		
Symptom experience						
Symptom severity						
T0	22.69 ± 10.29	8.00	.01	24.50 ± 10.46	.68	.71
T1	29.77 ± 12.31			21.10 ± 13.54		
T2	26.58 ± 12.29			18.20 ± 12.51		
Symptom hindrance						
T0	7.12 ± 6.67	13.01	.001	9.70 ± 5.54	.15	.92
T1	13.58 ± 9.66			8.90 ± 6.23		
T2	10.96 ± 8.72			8.40 ± 5.10		
Mental Health						
Depression						
T0	8.58 ± 6.65	11.40	.003	9.80 ± 8.99	5.70	.05
T1	10.62 ± 6.93			11.20 ± 8.18		
T2	10.04 ± 6.40			6.20 ± 6.49		
Anxiety						
T0	7.69 ± 6.04	4.95	.08	6.40 ± 5.29	2.10	.34
T1	9.62 ± 5.97			7.00 ± 5.46		
T2	7.77 ± 6.62			4.50 ± 3.54		
Stress						
T0	11.54 ± 7.27	4.93	.08	9.40 ± 7.72	.70	.70
T1	12.92 ± 6.63			9.80 ± 7.19		
T2	11.04 ± 6.99			6.40 ± 5.17		
MSPSS						
T0	2.45 ± .40	6.65	.03	2.31 ± .64	6.58	.03
T1	2.54 ± .35			2.43 ± .44		
T2	2.42 ± .40			2.41 ± .41		

*HRQoL* health-related quality of life, *T0* time 0, *T1*time 1, *T2*time 2, *MSPSS* Multidimensional Scale of Perceived Social Support

*P* value significant at two tailed

### Objective 3: Predictors of HRQoL of DAAs therapy users prior, during and at the end of therapy

At baseline, the developed model could significantly (*P* = .000) predict 37.5% (R^2^ = 39.8, AdjR^2^ = 37.5) the variance in the HRQoL prior to therapy. Depression and anxiety were significant variables that contributed to predict the change in HRQoL prior to therapy (41.3 & 29.1% respectively) (Table 7). All the other variables were excluded from the model as they could not significantly predict the HRQoL-T1: age, gender, PCR-T0, BMI-T0, MSPSS-T0 and stressT0.

**Table 7:** Predictors of HRQoL of DAAs therapy users prior, during and at the end of therapy

Model		Summary of the model			Unstandardized Coefficients	Standardized Coefficients			95.0% Confidence Interval for B		Collinearity Statistics	
		R	R^2^	AdjR^2^	B	Beta	t	Sig.	Lower Bound	Upper Bound	Tolerance	VIF
T0	(Constant)	.63	.39	.38	15.79		4.46	.000	8.69	22.89		
	Depression-T0				1.11	.41	3.13	.003	.39	1.83	.65	1.53
	Anxiety-T0				.98	.29	2.21	.032	.09	1.87	.65	1.53
T1	(Constant)	.88	.78	.76	18.13		1.45	.153	-6.95	43.21		
	Stress-T1				1.39	.44	4.04	.000	.70	2.09	.36	2.76
	HRQoL-T0				.37	.31	3.54	.001	.16	.58	.56	1.77
	Anxiety-T1				.91	.24	2.27	.028	.10	1.72	.37	2.66
	BMI-T1				-2.39	-.47	-2.82	.007	-4.11	-.69	.16	6.25
	BMI-T0				1.91	.35	2.12	.040	.09	3.730	.15	6.32
T2	(Constant)	.91	.83	.81	34.92		3.42	.002	13.97	55.87		
	HRQoL-T1				.68	.72	5.52	.000	.43	.93	.36	2.72
	Anxiety-T2				2.11	.66	5.27	.000	1.29	2.93	.46	2.17
	MSPSS-T2				-12.81	-.25	-3.09	.005	-21.33	-4.29	.93	1.06
	Anxiety-T1				-1.14	-.33	-2.11	.044	-2.25	-.03	.25	3.91

*HRQoL-T0* health-related quality of life time 0, *BMI-T1* body mass index time 1, *BMI-T0* body mass index time 0, *HRQoL-T1* health-related quality of life time 1, *MSPSS-T2* Multidimensional Scale of Perceived Social Support time 2

*P* value significant at two tailed

After 5/6 weeks of therapy (T1), the model significantly (*P* = .000) explained 76% (R^2^ = 78.2, AdjR^2^ = 76) the variance in the HRQoL. Out of 15 independent factors that entered the model, only HRQoLT0 & BMIT0 prior to therapy; and StressT1, Anxiety-T1 & BMI-T1 during therapy significantly explained the variation in HRQoL during the course of therapy. The five variables could significantly and prospectively predict 31.1, 35, 44.4, 24.4 & 46.5% of the variations in HRQoL during therapy (Table 7). All the other variables could not significantly predict the HRQoL during therapy: age, gender, PCR-T0, MSPSS-T0, MSPS-T1, depression-T0, anxiety-T0, stress-T0 and depressionT1, NDEXSCORE-T1, type of therapy.

At the end of therapy (T2), the model significantly (*P* = .000) predicted 80.5% (R^2^ = 83, AdjR^2^ = 80.5) of the variance in the HRQoL-T2. Out of 22 independent variables, four variables (HRQoL-T1, Anxiety-T1, Anxiety-T2, MSPSS-T2,) could significantly predict the variation in HRQoL. HRQoL & anxiety during the course of therapy and perceived support & anxiety at the end of therapy significantly and respectively predict 72.3, 33.1, 25.3 & 61.6% of the variations in HRQoL-T2 at the end of therapy (Table 7). All the other variables could not significantly predict the HRQoL-T2: depression-T2, stress-T2, HRQoL-T0, MSPSS-T0, MSPSS-T1, depression-T0, anxiety-T0, stress-T0, depression-T1, stress-T1, INDEXSCORE-T1, T1INDEXSCORE-T2, age, gender, disease duration, PCR-T1, BM-IT0 and MBI-T1.

The key finding of this study is that anxiety was always the constant variable that could significantly predict change in HRQoL during the three different time periods.

## Discussion

Healthcare consumers, whether patients or policy makers, are increasingly interested in how medical intervention impacts PROs, such as patient’s HRQoL [33–36]. Therefore, several studies examined the HRQoL among HCV on antiviral therapy; and agreed that Peg-IFN and ribavirin therapy of chronic HCV remains problematic, as it causes unpleasant side effects that could affect the patients’ HRQoL [34, 35]. Therefore, a new DAAs therapy has been developed and has been found to be a well-tolerated therapy with low adverse side effects [10, 13]. Although the benefits of DAAs therapy are well established; its effects on HRQoL are less certain [10, 37]. Therefore, this prospective observational study is the first study to evaluate a number of PROs, including HRQoL, mental health status (i.e. depression, anxiety and stress), and perceived social support among Egyptian HCV patients, while receiving DAAs therapy; either a dual or triple therapy. Consequently, this section provides an interpretation of the study’s results based on the stated three aims, implications for nursing practice and suggested recommendations for future research and limitations of the study.

This study recruited 62 patients with HCV who were treated with DAAs. Our study provides several new and important lines of evidence about these patients’ HRQoL, symptom experience, mental health and perceived social support of patients receiving DAAs (Dual and triple therapy) that will enhance health care providers’ insight about these patients’ needs during the course of therapy.

### Objective 1: PROs of HCV patients receiving DAAs therapy prior, during and at the end of therapy

Our study clearly documented the effect of time on HRQoL, symptom experience, mental health and perceived social support. There was a significant effect for time on HRQoL; it was significantly poorer after 5/6 weeks (peak point of impairment) of therapy than at the baseline and at the end of therapy. It is interesting that the HRQoL score significantly increased from T0 to T1, indicting impairment of HRQoL and then backed to baseline score. This finding is very important as it confirms the need for follow up and tracking of the HRQoL among patients on antiviral therapy. Previously, it was ascertained that in addition to the baseline impairment of HRQoL in patients with chronic HCV, treatment regimens can impose additional PROs burdens [38]. This impairment is further amplified by the antiviral (peg-IFN and RBV) side effects, particularly anemia and depression. Similarly, non cirrhotic patients who received triple therapy had significant impairments in HRQoL using Short Form (SF)-36. Dual therapy was also associated with moderate HRQoL and work productivity impairment regardless of the stage of fibrosis for role physical and role emotional of SF-36, but this impairment was significantly lower when compared to triple therapy [10]. Younossi et al [10]. showed that at week 4 of active treatment with DAAs therapy (Ledipasvir and Sofosbuvir), a significant decline in some domains of HRQoL was observed in patients without and with mild fibrosis, including physical and social functioning, role physical and emotional, and vitality of SF-36, physical and functional well-being and fatigue, and activity/energy domain of Chronic Liver Disease Questionnaire-HCV (CLDQ-HCV).

However, our study showed that by the end of therapy the scores of HRQoL, mental health and symptom experience were almost back to baseline score. A previous study on other hand showed that by the end of treatment, a more substantial deterioration was observed in most of the HRQoL domains including role physical and role emotional of SF-36 regardless of patients’ fibrosis status [10]. Furthermore, at the end of treatment, perceived general health, emotional well-being, and worry domains significantly improved in both fibrosis cohorts. At follow-up, all HRQL domains and work productivity returned to their baseline levels or moderately improved as early as post-treatment week 4 [10].

Time also significantly impacted on symptom experience (severity & hindrance) and mental health (depression & anxiety). Symptom hindrance and mental health were significantly poorer at T1 (5/6 weeks of therapy) than at baseline (T0). Additionally, anxiety and stress were significantly higher during the treatment period (T1) than at the end of treatment (T2). Interestingly, anxiety at baseline, after 5/6 weeks and at the end of therapy, was significantly associated with severity and hindrance of symptoms and HRQoL at different points in time (*p* < 0.02, r ranged from 0.38 to 0.79). Previously, it was ascertained that in addition to the baseline impairment of mental health of patients with chronic HCV, treatment regimens can impose additional mental health impairment [25]. Therefore, it was unsurprising to find perceived social support significantly higher during the treatment period than at baseline (T0) and end of therapy (T2). For that reason, more healthcare support is required during the course of therapy, particularly to improve HRQoL, symptom experience and mental health, which have been found to significantly worsen during the course of therapy.

### Comparison of basic demographic and medical data between dual and triple users

For the purpose of comparing the dual vs. the triple therapy users, the baseline demographic and medical characteristics of the two groups of participants were initially compared. We found that the dual participants were less educated, more likely to live in urban areas and more cirrhotic than triple users. At the time of data collection in Egypt, two treatment regimens were delivered according to the guideline protocol of the National Committee of Control HCV (NCCVH) for patients with HCV. According to this guideline, patients with more advanced liver disease were always ineligible for peg-IFN treatment and thus dual therapy was presented to them. This could help explain our findings, which also confirm that our sample was representative of HCV patients who received DAAs therapy at that time.

### Comparison of HRQoL, mental health and symptom experience between dual and triple users

Similar to an earlier study [36], we found that prior to therapy HRQoL was not significantly different between dual and triple users. Also, mental health status, symptom experience and perceived support were similar. However the triple users were more likely to experience more deterioration in their HRQoL and higher levels of anxiety during the course of therapy. These findings might be due to the fact that the effect of Peg-IFN on patients’ HRQoL and anxiety was found to be higher than the effect of a Peg-IFN-free regimen. However, the adherence score was high and similar in dual and triple users, indicating the effect of treatment on HRQoL and anxiety. It is well established that HCV infection is associated with poorer HRQoL and a part of impaired health of these patients is related to comorbid psychiatric disorders and interferon treatment. Interferon treatment is an important cause of depression and anxiety in HCV patients and is sometimes associated with irritability, manic episodes, or acute confusional state [39]. Interferon is associated significantly with increased somatic but not cognitive affective symptoms of depression and with increased anxiety and fatigue during treatment [40].

At the end of therapy, the perceived support was not significantly different among dual and triple users. However, there was a significant effect of time on changing the score of perceived social support, where dual and triple users reported higher perceived support during the course of therapy than in baseline and end of therapy. This finding was similar to previous studies that reported that with increasing disease severity there is increased family support [29, 41].

By the end of therapy, it was observed that the mean score of HRQoL, symptom severity and hindrance, depression, anxiety and stress, had improved in the two arms of therapy, with no significant difference between them. On the other hand, in the only recent large identified study, fatigue, HRQoL using a disease specific CLDQ-HCV and generic SF-36 and work productivity were examined between patients receiving the triple therapy and patients receiving dual therapy [25]. It was found that patients receiving the triple therapy (*n* = 327) experienced poorer HRQoL and work productivity than patients receiving the dual therapy (*n* = 201) (*p* ≤ 0.01) at the end of treatment [25]. Interferon in general has a negative impact on patients’ HRQoL during the course of therapy and the potential low adherence to the treatment regimen was confirmed [42, 43]. Otherwise, our study found no significant difference between the two regimens, although the dual therapy had a minimal negative impact on patients’ HRQoL compared to the triple therapy. Our explanation is that the shorter duration of the treatment course (24/12 weeks according to the regimen type) may have been related to the lower treatment-related PROs burden of these patients and therefore enhancing their adherence level to medication would be expected [25]. However, it may also be due to the difference in methodology used between our study and Younossi’s study [25] in terms of questionnaires used and time point measurements. Also, the sample size was smaller than previous studies, which might not be helpful in finding a significant difference at the end of therapy between the two groups of therapy.

### Objective 2: PROs of dual users versus triple users prior, during and at the end of treatment

A comparison of the pattern of change in PROs among triple and dual therapy users through the three time periods revealed that triple therapy users were significantly more likely to report poorer HRQoL, symptom severity, symptom hindrance and depression, and increase in perceived social support score at T1 than at baseline. In contrast, dual users had no significant change in their HRQoL, symptom experience and mental health, while their perceived social support was significantly increased during T1 in comparing to T0 and T2 like triple therapy users. This finding indicates that whatever the type of therapy, patients reported a high perceived support from their spouse, family and friends. Culturally this finding is unsurprising as social support increases during illness. Previously, interferon-free regimens have a modest negative effect on PROs whatever the disease stage [44].

### Objective 3: Predictors of HRQoL of DAAs therapy users prior, during and at the end of therapy

A number of multivariate analyses were run to identify the independent predictors of HRQoL at the three different points in time. At baseline, the eight factors in the model significantly explained 37.5% of the variance in overall HRQoL prior to therapy. However, only anxiety and depression were significant variables, which appear to be the key determinants for HRQoL prior to therapy. Similar to a previous study [25], our study showed that pre-treatment depression and anxiety are the major factors associated with impaired HRQoL among patients with HCV before initiation of treatment. The high prevalence of depression, stress and anxiety in patients with HCV before the initiation of treatment has previously been reported [36]. Using the backwards multivariate linear regression, Bonkovsky et al [33] found that depression and anxiety [using the Beck Depression Inventory (BDI)] were significantly associated with impaired HRQoL summary scores (using the SF-36). It was also reported that the severity of depressive symptoms was highly correlated with fatigue severity, functional disability and somatization [45]. Baseline depression, anxiety, treatment-related adverse events and cirrhosis have been found to be the most consistent independent predictors of disease specific quality of life using the CLDQ-HCV at all points in time [25, 36]. In patients with cirrhosis who were treated with dual or triple therapy, the multivariate analysis at baseline showed that, being female, baseline depression, anxiety, insomnia, fatigue, a history of unsuccessful treatment, and having cirrhosis were associated with more impairment in PROs (i.e. Functional Assessment of Chronic Illness Therapy-Fatigue, CLDQ-HCV, Work Productivity and Activity Impairment Questionnaire: Specific Health Problem [WPAI-SHP]) scores. Furthermore, during treatment, receiving an IFN-containing regimen was another independent predictor of PROs impairment, whereas having cirrhosis was no longer associated with any of the PROs impairment during treatment [44].

Accordingly, whatever the liver disease stage and the instruments that were used to examine the association between mental health status and HRQoL, it has been found that depression and anxiety were significantly associated with HRQoL of patients with HCV. Therefore, the high prevalence of depression and anxiety symptoms among HCV patients not receiving antiviral therapy have justified the importance of regular psychosocial screening and support for them independent of antiviral therapy [45]. It is important to keep in mind that, prior to the initiation of treatment, patients with HCV appeared to experience impairment of their HRQoL [38]. In a cross-sectional study of 81 HCV-infected patients who were not receiving antiviral therapy, anxiety, depression, psychopathological symptoms, social support and resilience were assessed [41]. It was found that depression and anxiety scores were significantly higher among HCV patients than in a healthy control group [41] as 62.9% of HCV patients had a major depressive disorder diagnosis, and 42.3% had significant depressive symptoms, according to the BDI-II [46].

After 5 to 6 weeks of therapy (T1), the model significantly explained 76% of the variance in the HRQoL. HRQoL T0 & BMI T0 prior to therapy; and Stress T1, Anxiety T1 & BMI T1 during therapy significantly explained the variation in HRQoL during the course of therapy. These findings give support to the important role that these factors play in HRQoL of HCV patients during therapy. These results were previously unknown; therefore, it was difficult to compare our study’s findings with previous studies. In patients with cirrhosis who were treated with dual or triple therapy, the multivariate analysis during treatment (4 weeks after starting therapy) showed that being female, baseline depression, anxiety, insomnia, fatigue, and having a history of unsuccessful treatment, and receiving an IFN-containing regimen were the predictors of PROs impairment [Functional Assessment of Chronic Illness Therapy-Fatigue, CLDQ-HCV, WPAI-SHP]), whereas having cirrhosis was not associated with any of the PROs impairment during treatment [44].

Based on our study’s findings, we recommend that baseline HRQoL and BMI should be systematically assessed before starting the antiviral therapy for early detection and the improvement of the impairment before the initiation of therapy. Also, the patients’ anxiety, stress and BMI should be frequently assessed and followed up through the course of antiviral therapy. Additionally, HRQoL & anxiety during the course of therapy and perceived support & anxiety at the end of therapy significantly and respectively predict the variations in HRQoL T2 at the end of therapy. A pervious study found that a history of pre-treatment anxiety, depression, fatigue, female gender, and presence of cirrhosis were major predictors of disease specific quality of life impairment [25].

Additionally, our results confirmed previously reported data that anxiety is one of the major constant predictors of HRQoL impairment (at different time points) [25]. Therefore, health care providers should develop a supportive care program to help decrease anxiety levels that might later impact on these patients HRQoL. Studying this relationship in these patients is highly recommended. Also, a comprehensive care plan including all these associated factors is urgently needed to avoid the deterioration of patients’ HRQoL during the course of therapy. Also, an exploration of the causes of increased anxiety among HCV patients at baseline and during the course of antiviral therapy is required.

### Comparison between males and females

We found that females had a higher depression level and an increased impaired HRQoL than males prior to treatment even after controlling for age, PCR T0, BMI T0 and duration of disease in months. A comparison between male and female patients revealed that our findings were similar to a previous study [36]. Bonkovsky et al. [33], using a multivariate model, found that female gender, greater BMI, older age, current cigarette smoking, a higher depression score, and use of antidepressant or anxiolytic medications at baseline were significant predictors of poor HRQoL, particularly of the physical summary score among HCV patients [33]. However, at the end of treatment, our study did not agree with the findings of a previous study [36] which found that females had poorer PRQs than males at the end of treatment.

Interestingly, although females had a significantly lower PCR (viral load) than males prior to therapy, they experienced poorer HRQoL, higher depression, higher symptom severity and hindrance of symptoms. Similarly, Younossi et al (2014) [36] found that females, without considering the viral load, showed more impairment in PROs than males; including physical components of SF-36, physical well-being, fatigue, systematic domains of CLDQ-HCV and activity impairment. Clearly, further research is required to investigate this further.

Nurses and healthcare providers should therefore care for these female patients, particularly their mental health status and depression symptoms, by developing intervention programs that aim to improve their mental health status, which will be reflected on their HRQoL and their overall life. Due to the reduced sample size at time points two and three (T1 & T2), there is an urgent need to repeat this study, but using a larger balanced sample of male and female patients in Egypt and elsewhere.

### Recommendations

Anxiety has been found to be high in patients receiving HCV antiviral therapy, particularly those following triple therapy. Consequently, we highly recommend that anxiety among these patients be systematically researched and analysed and a nursing intervention plan be developed to support these individuals. The patient’s perspective in terms of PROs (i.e. HRQoL, mental health and perceived social support) must be considered by nurses and healthcare providers regularly in the plan of care to improve treatment experience by decreasing the treatment burden.

A symptom management program should be developed and delivered by highly qualified and well trained nurses to HCV-patients who attend regular consultations during the antiviral therapy. Also, a phone number should be available for delivering symptom management advice at any time and at any place according to the patient’s needs and without interrupting the health care providers’ work. Designing simple illustrated educational materials as guidance would be helpful to answer some of these patients’ questions and explain the treatments adverse effects as well as how to overcome them during the course of therapy.

Although this study used a quantitative approach, some patients gave qualitative comments to “explain” their answer, which increased the understanding of patients’ suffering as well as their needs. Therefore, a qualitative approach is recommended to explore psychosocial needs and to suggest self-care approaches that can help these patients to overcome the medications adverse effects as well as symptom hindrance.

Face to face interviews were used to complete the instruments, as most of the patients were uneducated. This approach was time consuming to the researcher as well as to the participants. Thus, it was appropriate to use only a disease specific HRQoL questionnaire (i.e. LDSI-2.0) alongside the other instruments. Using a disease specific HRQoL questionnaire was helpful in identifying the unique symptoms experienced by patients on antiviral therapy. Subsequently, we recommend that future studies demonstrate both generic and disease specific HRQoL measurements to gain more insights into patterns of change in various domains of HRQoL among these patients, and which domains are more likely to be affected during the course of therapy.

Health-related quality of life is a complex concept with numerous dimensions. It should be an important outcome measure for all persons with HCV generally, and on antiviral therapy specifically, to ensure that the healthcare resources and medical treatment, as well as nursing interventions offered to this population are providing an improvement in patient’s daily activities and well-being. Ascertaining HRQoL requires engaging the patients in their plan of treatment and intervention, as this cannot be ascertained independently by a clinician or the nurse.

### Potential limitations

The fact that patients know whether they have responded to treatment when they complete the HRQOL instruments, has been considered a potential confounder in most longitudinal studies of HRQOL in chronic hepatitis C [33]. However, in this study we could not control patients’ and health care providers’ blindness to the PCR results at the time of data collection. Therefore, some of the patients did know their PCR results during data collection, which may have affected the patients’ anxiety level as well as their HRQoL.

The participants were recruited from one centre largely due to the resources available to the research team. It was felt however, that a representative sample was obtained, as all the other centres were very similar in patient demographics, as well as clinic resources, policies and procedures. All these centres follow the same set of national guidelines for the treatment of patients with chronic HCV and are supervised by the NCCVH.

The limited sample size may have been one of the reasons that we found no significant association between some of the studied variables. Replicating this study with a larger sample of patients is needed to establish the reliability of these results.

Taking into consideration the real life nature of the treatment, losses in the availability of laboratory results during treatment and follow up did occur, which made it difficult to keep data consistency. Therefore, a future study should consider the haemoglobin levels during the follow up of the HRQoL.

## Conclusions

This study found a significant change in HRQoL across the three different time periods among patients receiving DAAs. Only perceived support at baseline and anxiety during treatment were significantly higher among the triple therapy group than dual group when comparing the three different time points. At baseline, depression was the main variable that contributed to predicting change in HRQoL prior to therapy. During therapy, stress-T1, body mass index (BMI)-T1 and HRQoL-T0 significantly and respectively predicted HRQoL-T1. At the end of therapy, HRQoL-T1 and anxiety-T2 significantly predicted the variation in HRQoL-T2.

This study’s findings highlight the critical importance of assessing HRQoL, mental health and perceived social support in patients receiving HCV antiviral therapy. These study’s findings add value to what is important to healthcare providers by including outcomes from the patients’ perspective. Therefore, the patient’s perspective in terms of HRQoL, mental health and perceived social support should be fully considered by nurses and healthcare providers when planning care.

## Abbreviations

BDI: Beck depression inventory
BMI: Body mass index
CASE-Index: Center for adherence support evaluation index
DAAs: Direct acting antivirals
DASS-21: The depression anxiety stress scale
HCV: Hepatitis C virus
HRQoL: Health-related quality of life
INDEXSCORE: Index score for the center for adherence support evaluation index
LDSI-2.0: The liver disease symptom index-2.0
MSPSS: The multidimensional scale of perceived social support
NHTMRI: National Hepatology and Tropical Medicine Research Institute
PCR: Polymerase Chain Reaction
Peg-IFN: Pegylated interferon
PROs: Patient reported outcomes
T0: Time 0 for data collection before/baseline data
T1: Time 1 after 4/5 weeks after therapy
T2: Time 2 at the end of therapy
WPAI-SHP: Work Productivity and Activity Impairment Questionnaire: Specific Health Problem
